# Existence of multiple organ aging in animal model of emphysema induced by cigarette smoke extract

**DOI:** 10.18332/tid/143853

**Published:** 2022-01-17

**Authors:** Guibin Liang, Zhihui He, Yan Chen, Hongbo Zhang, Huaihuai Peng, Dandan Zong, Yingjiao Long

**Affiliations:** 1 Department of Critical Care Medicine, The Third Xiangya Hospital, Central South University, Changsha, China; 2 Department of Respiratory Medicine, The Second Xiangya Hospital, Central South University, Changsha, China; 3 Department of Pathology, The Second Xiangya Hospital, Central South University, Changsha, China; 4 Department of Intensive Care Unit, The Second Xiangya Hospital, Central South University, Changsha, China

**Keywords:** cigarette smoke, decitabine, emphysema, multiple organs, aging

## Abstract

**INTRODUCTION:**

It is commonly considered that COPD or at least emphysema represents accelerated lung aging induced in part by oxidative damage from cigarette smoke components. However, the issue if there are any aging signs in other organs in patients with COPD or emphysema remains unclear. The aim of this study is to explore whether there is multiple organ aging in the animal model of emphysema induced by cigarette smoke extract (CSE), and to ascertain the possible mechanisms, if any.

**METHODS:**

The animal model of emphysema was induced by CSE. Histomorphological changes in lung, heart, liver, kidney and spleen tissues were measured after staining with hematoxylin and eosin (H&E). The concentrations of stem cell factor (SCF), CyclinD1 and superoxide dismutase (SOD) in serum were determined by ELISA kit. The expressions of p16 (INK4a), Sca-1, eNOS proteins and mRNA in lung, heart, liver, kidney and spleen tissues were detected by Western blotting and quantitative reverse transcriptase polymerase chain reaction (qRT-PCR), respectively. Decitabine (Dec) was applied to examine whether it could alter the changes caused by CSE.

**RESULTS:**

The histomorphology of lung tissue was significantly changed, while other organs exhibited normal structure and histomorphology. The concentrations of SCF, CyclinD1 and SOD in serum were lower in the CSE group than in the control group. The expression levels of p16(INK4a) protein and mRNA in lung, heart, liver, kidney and spleen tissues were higher in the CSE group than in the control group, while the expression levels of Sca-1 and eNOS proteins and mRNA were lower in the CSE group than in the control group, in the tissues described above. Dec could partly alleviate the damages caused by CSE and the degree of alleviation resulted by Dec varied from organ to organ.

**CONCLUSIONS:**

In addition to the aging of the lung tissue in the emphysema animal model induced by CSE, the tissues of the heart, liver, kidney and spleen were also in the progress of aging, but the sensibility and affinity of lung to CSE were higher than those of the other organs. Multiple organ aging may also exist in the animal model of emphysema induced by CSE. DEC can partly alleviate the multiple organ aging caused by CSE.

## INTRODUCTION

Aging is characterized by progressive decline in tissue and organ function and increased risk of mortality, which is associated with a wide range of human disorders, including cancer, diabetes, cardiovascular, and neurodegenerative diseases. Some studies have shown that chronic obstructive pulmonary disease (COPD) or at least emphysema was a disease of accelerated lung aging^1^. Environmental gases, such as cigarette smoke or other pollutants, may accelerate the aging of lungs or worsen aging-related events in lungs by causing defective resolution of inflammation, for example, by reducing antiaging molecules, such as histone deacetylases, and this consequently induces accelerated progression of COPD. There are many aging-associated changes in the lungs, including decreased size of thoracic cavity, limiting lung volume. Our previous studies demonstrated that the expression level of p16(INK4a) was increased, and the expression level of stem cell antigen-1 (Sca-1) was decreased in lung tissue of emphysema^2,3^. However, the issue if there are any aging signs in other organs of the body, such as heart, liver, kidney and spleen, with COPD or emphysema, remains unclear. For ethical reasons, we cannot carry out experiments on organ aging in patients with COPD, and so we employed an animal model. Smoking is an independent risk factor for cardiovascular and lung diseases. The animal model of emphysema induced by intraperitoneal injection of cigarette smoke extract (CSE) has been verified in previous studies, and it is equivalent to the animal model of emphysema induced by cigarette smoke (CS) exposure in lung function and histomorphology^4^. CSE contains more than 4000 chemicals found in CS, including free radicals, toxins, and electrophiles. An increasing number of experiments have shown that CS or CSE could accelerate the senescence of leukocyte, endothelial progenitor cell (EPC) and lung tissue and reduce their regeneration ability^3^. It is commonly considered that COPD or at least emphysema represents accelerated lung aging induced in part by oxidative damage from cigarette smoke components.

There are many indicators that can be used to assess the function of the lung, heart, liver and kidney in humans, but not in mice. The main inflammatory cells involved in the inflammatory response of COPD are neutrophils, macrophages, and T lymphocytes. The mechanisms of their action are mainly through the release of neutrophilic proteases, including elastase, cathepsin G and matrix protease that cause a chronic mucus hypersecretion state and destroy lung parenchyma. In the aging process, the long-term stimulation of pro-inflammatory cytokines such as TNF-α, IL-1β and IL-6 in the body can lead to a chronic, low-degree and mild inflammatory response, thus causing or increasing the occurrence of age-related degenerative diseases. In this study, the potential aging of organs in mice was evaluated by the detection of stem cell factor (SCF), Cyclin D1, superoxide dismutase (SOD), p16(INK4a), stem cell antigen-1(Sca-1), and endothelial nitric oxide synthase (eNOS), which are linked to oxidation, apoptosis and aging in humans and animals^5^. SCF promotes the proliferation and differentiation of hematopoietic cells and regulates the growth and development of hematopoietic cells. Cyclin D1 acts on G1 phase and interacts with many proteins to promote cells to enter S phase. SOD could eliminate excess oxygen free radicals and their derivatives so as to protect cells from damage and maintain normal metabolism. The antibody p16(INK4a) is one of the best aging biomarkers and is suppressed in early embryogenesis and progressively induced during aging. Sca-1 is usually associated with stem cells or progenitor cells proliferation and self-renewal, and is a marker of youth. eNOS is the key enzyme that catalyzes the production of NO, an endogenous signal molecule which maintains vascular homeostasis, including blood pressure homeostasis, vascular permeability, tension regulation and hypoxic compensatory mechanism. Decitabine (Dec) is a widely used DNA methylation inhibitor, which triggers demethylation *in vitro* and *in vivo*, resulting in continuous reactivation of epigenetic silencing genes. In our previous study^3^, it was demonstrated that Dec alleviated lung senescence in a CSE-induced emphysema animal model. In the present study, Dec was applied to identify whether it can alleviate the aging of other organs, if any.

## METHODS

In the present study, we follow the methods of He et al.^3^.

### Animals

Forty male C57BL/6J mice aged 6 weeks were randomly enrolled in this study and fed in a cleaning unit at 23–25^o^C, 50–60% humidity, with a 12-hours rhythm of night and day. All animals were purchased from Shanghai laboratory animal center of the Chinese Academy of Sciences (SLACCAS, Shanghai, China). The study was approved by the Institutional Review Board of Central South University and conformed to the guiding principles for research involving animals and human beings^6^.

### Preparation of CSE and decitabine

CSE was prepared according to a previously published method^2^. Briefly, one Fu-Rong cigarette (Tar: 13 mg, Nicotine: 1.0 mg, carbon monoxide: 14 mg/cigarette; China Tobacco Hunan Industrial, Changsha, China) was burned and the smoke passed through 4 mL of phosphate-buffered saline (PBS) via a vacuum pump at a constant pressure of -0.1 kPa. This product was further filtered through a filter with 0.22 μm pores (Thermo Fisher Scientific, Waltham, MA, USA) to remove particles and bacteria. The solution was prepared freshly for each experiment. Five grams of decitabine (Sigma, MO, USA) was dissolved in 2mL PBS and further diluted to 25 mg/mL, sub-packaged and stored under -80^o^C until the experiments.

### Experimental protocol

The mouse emphysema model was established as previously described^3^. The C57BL/6J mice were divided into four groups: control, Dec, CSE, and CSE+Dec (10 per group). The total experimental period was four weeks, with intraperitoneal injection of PBS, Dec, or CSE (Table 1). The doses of intraperitoneal injection of PBS, Dec, CSE were 0.3 mL/20 g, 0.3 mL/20 g, 2.5 mg/kg (0.3 mL/20 g constant volume), respectively. At Day 28, the mice were disposed for the experiments.

**Table 1:** Experiment schedule (N=10)

*Day*	*0*	*11*	*15*	*17*	*19*	*22*
Control	PBS	PBS	PBS	PBS	PBS	PBS
Dec	PBS	PBS	Dec	Dec	Dec	PBS
CSE	CSE	CSE	PBS	PBS	PBS	CSE
CSE+Dec	CSE	CSE	Dec	Dec	Dec	CSE

All treatments were conducted by intraperitoneal injection. All animals were disposed on Day 28 after the start of treatment. Dec: decitabine. CSE: cigarette smoke extract. PBS: phosphate buffered saline.

### Measurement of the concentrations of SCF, Cyclin-D1 and SOD in serum

The mouse was weighed and anesthetized by intraperitoneal injection of 10% chloral hydrate (3 mL/kg body weight). Then the blood was sampled by cutting the tail and centrifuged within an hour at 700g for 10 min. The supernatant was collected and stored at -40°C. The concentrations of SCF, Cyclin D1 and SOD in serum were measured with ELISA kits (R&D systems, USA) according to the manufacturer’s instructions.

### Slice staining and identification of emphysema

After blood collection, the mice were sacrificed by an overdose of anesthetic. Lung, heart, liver, kidney and spleen slices were stained with hematoxylin and eosin (H&E) (Sigma, USA). Emphysema was quantified based on the measurement of the mean linear intercept (MLI) and destructive index (DI). Briefly, MLI was measured by dividing the length of a line drawn across the lung section by the total number of intercepts. The DI was calculated by dividing the defined destructive alveoli by the total number of alveoli. Destructive alveolus was defined as having at least one of the following: alveolar wall defects; intraluminal parenchymal rags in alveolar ducts; obvious abnormal morphology; and typical emphysematous changes. The analysis was performed using a microscopic point-count technique at ×200 magnification. The histomorphology assessment was performed in a blinded manner.

### Western blot analysis

Briefly, protein was mixed 1:1 with 2×SDS loading buffer and incubated at 100°C for 4 min. Then, the protein was electrophoresed in 10–12% SDS-polyacrylamide gel and transferred electrophoretically onto a polyvinylidene difluoride microporous membrane (Millipore, Billerica, MA, USA). Membranes were incubated with primary antibody overnight [p16(INK4a): 1:200, Santa Cruz Biotechnology, USA; Sca-1: 1:200, Biolegend, USA; eNOS: 1:1000, Proteintech, USA), and then washed carefully with Tris-buffered-saline with Tween-20 (TBST) followed by re-incubation with secondary antibodies (1:3000; 1 h). After washing with TBS-T again, immunoreactive bands were detected using ECL chemiluminescent substrate (Thermo, USA).

### Quantitative RT-PCR

Total RNA was extracted from tissues using Trizol reagent (Invitrogen, USA). First-strand cDNA was synthesized using the RevertAid™ First Strand cDNA Synthesis Kit (Fermentas, USA) according to the manufacturer’s instructions, and used as the template for quantitative RT-PCR analysis. Gene expression and analysis were performed by RT-PCR using SYBR Green qPCR Master Mix (Applied Biosystems, USA) on Bio-rad CFX96 Real Time system (Bio-Rad, USA) with β-actin used as an internal control. The ΔΔCt method was adopted to quantify the fold change of expression levels. Each experiment was repeated two times in triplicate. The primers sequences used were as follows: β-actin, 5’-CATCCTGCGTCTGGACCTGG-3’ (forward), 5’-TAATGTCACGCACGATTTCC-3’ (reverse); p16(INK4a), 5’-CCGCCTCAGCCCGCCTTTTT-3’ (forward), and 5’-CCGCCGCCTTCGCTCAGTTT-3’ (reverse); Sca-1, 5’-ACACCGAGCCCAGGTAACCC-3’ (forward), and 5’-CTGGTCCGCTCAGGACAGCA -3’ (reverse); eNOS, 5’-GGGCTCGGGCTGGGTTTAGG-3’ (forward), and 5’-CCTGGGCACTGAGGGTGTCG-3’ (reverse).

### Statistical analysis

Each experiment was repeated three times. Analyses were performed using SPSS for Windows 16.0 (SPSS Inc., Chicago, IL, USA). All data were expressed as mean ± standard deviation (SD). Analysis of differences among groups were performed using analysis of variance (one-way ANOVA), followed by *post hoc* analysis as appropriate. Values of p<0.05 were considered statistically significant.

## RESULTS

### Weight change of mouse

On Day 0, the body weight (g) of mice in the control, Dec, CSE+Dec and CSE groups were 16.60±1.64, 17.00±1.92, 15.86±1.45 and 16.15±1.80, respectively, and there was no statistical difference between groups (p>0.05). On Day 28, the body weight (g) of mice in the control, Dec, CSE+Dec and CSE groups were 24.14±0.84, 23.36±0.79, 23.14±1.20 and 24.13±2.27, respectively, and there was no significant difference between the two groups (p>0.05) (Table 2).

**Table 2:** Weight change of mouse

	*Day 0 Mean ± SD*	*Day 28 Mean ± SD*
Control	16.60 ± 1.64	24.14 ± 0.84
Dec	17.00 ± 1.92	23.36 ± 0.79
CSE+Dec	15.86 ± 1.45	23.14 ± 1.20
CSE	16.15 ± 1.80	24.13 ± 2.27
F	0.681	1.065
p	0.571	0.380

Dec: decitabine. CSE: cigarette smoke extract.

### Lung, heart, liver, kidney and spleen histomorphology

**Figure 1:**
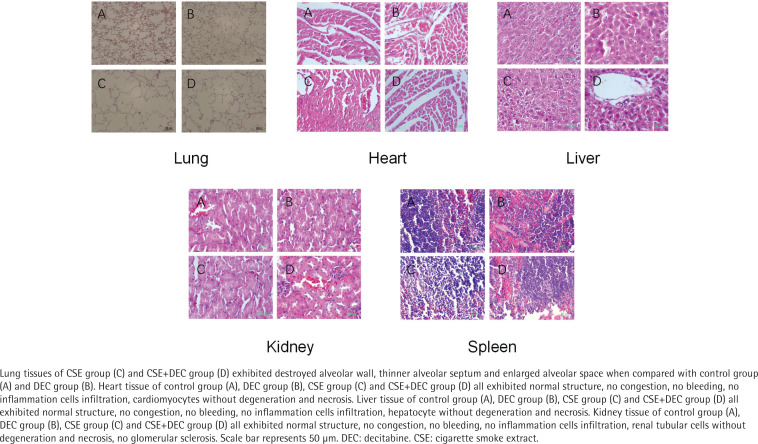
Histomorphological changes of lung, heart, liver, kidney and spleen tissues

As shown in Figure 1, the lung tissues of CSE group (Figure 1, Lung C) and CSE+Dec group (Figure 1, Lung D) exhibited damaged alveolar walls, thinner alveolar septa and enlarged alveolar spaces when compared with the control group (Figure 1, Lung A) and Dec group (Figure 1, Lung B). Quantitative analysis showed that MLI and DI were significantly higher in the CSE group (69.65 ± 10.19 μm, 45.36 ± 6.96%) and CSE+Dec group (53.97 ± 8.86 μm, 35.83 ± 5.75%) than those in the control group (27.69 ± 5.37 μm, 9.95 ± 1.19%) and Dec group (29.17 ± 5.31 μm, 10.36 ± 1.40%) (p<0.01), respectively. Furthermore, MLI and DI in the CSE group were significantly higher than those in the CSE+Dec group (p<0.01 or p<0.05), respectively. There was no significant difference between the control group and Dec group in MLI or DI (p>0.05) (Table 3).

**Table 3:** Lung histomorphology (N=10)

	*MLI (μm) Mean ± SD*	*DI (%) Mean ± SD*
Control	27.69 ± 5.37	9.95 ± 1.19
Dec	29.17 ± 5.31	10.36 ± 1.40
CSE	69.65 ± 10.19^a,b^	45.36 ± 6.94^a,b^
CSE+Dec	53.97 ± 8.86^a,b,d^	35.83 ± 5.75^a,b,c^

Dec: decitabine. CSE: cigarette smoke extract. MLI: mean linear intercept. DI: destructive index.

a p<0.01 vs Control;

b p<0.01 vs Dec;

c p<0.01 vs CSE;

d p<0.01 vs CSE.

As shown in Figure 1, the heart tissue of the control group (Figure 1, Heart A), Dec group (Figure 1, Heart B), CSE group (Figure 1, Heart C) and CSE+Dec group (Figure 1, Heart D) all exhibited normal structure, no congestion, no bleeding, no inflammation cells infiltration, cardiomyocytes without degeneration and necrosis.

As shown in Figure 1, the liver tissue of the control group (Figure 1, Liver A), Dec group (Figure 1, Liver B), CSE group (Figure 1, Liver C) and CSE+Dec group (Figure 1, Liver D) all exhibited normal structure, no congestion, no bleeding, no inflammation cells infiltration, hepatocytes without degeneration and necrosis.

As shown in Figure 1, the kidney tissue of control group (Figure 1, Kidney A), Dec group (Figure 1, Kidney B), CSE group (Figure 1, Kidney C) and CSE+Dec group (Figure 1, Kidney D) all exhibited normal structure, no congestion, no bleeding, no inflammation cells infiltration, renal tubular cells without degeneration and necrosis, and no glomerular sclerosis.

As shown in Figure 1, the spleen tissue of control group (Figure 1, Spleen A), Dec group (Figure 1, Spleen B), CSE group (Figure 1, Spleen C) and CSE+Dec group (Figure 1, Spleen D) all exhibited normal structure, splenic nodule presenting, and no congestion in splenic sinus.

### Concentrations of SCF, Cyclin D1 and SOD in serum

**Figure 2:**
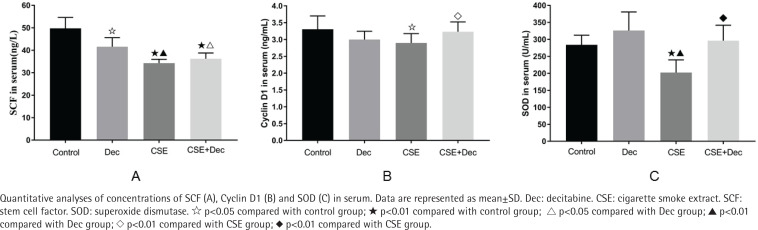
Concentrations of SCF, Cyclin D1 and SOD in serum

The concentration of SCF in serum was significantly lower in the Dec group (41.56 ± 4.04 ng/L), CSE group (34.30 ± 1.67 ng/L) and CSE+Dec group (36.23 ± 2.56 ng/L) than in the control group (49.75 ± 4.85 ng/L) (p<0.05 or p<0.01). The concentration of SCF in serum was significantly lower in the CSE group and CSE+Dec group than in the Dec group (p<0.05 or p<0.01). However, there was no difference in the concentration of SCF in serum between the CSE group and CSE+Dec group (p>0.05) (Figure 2 A).

The concentration of Cyclin D1 in serum was significantly lower in the CSE group (2.90 ± 0.28 ng/mL) than those in the control group (3.30 ± 0.40 ng/mL) and CSE+Dec group (3.23 ± 0.29 ng/ mL) (p<0.05 or p<0.01). There was no difference in the concentration of Cyclin D1 in serum among the control group, Dec group (3.00 ± 0.24 ng/mL) and CSE+Dec group (p>0.05) (Figure 2 B).

The concentration of SOD in serum was significantly lower in the CSE group (202.31 ± 37.40 U/mL) than those in the control group (284.08 ± 28.13 U/mL), Dec group (326.19 ± 54.73 U/mL) and CSE+Dec group (296.20 ± 45.43 U/mL) (p<0.01). There was no difference in the concentration of SOD in serum among the control group, Dec group and CSE+Dec group (p>0.05) (Figure 2 C).

### Expression levels of p16(INK4a) protein and mRNA in lung, heart, liver, kidney and spleen tissues

**Figure 3:**
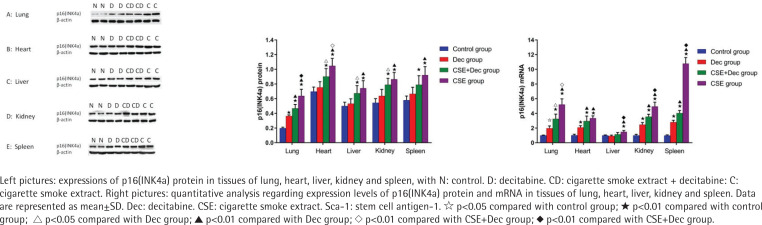
Expression levels of p16(INK4a) protein and mRNA in lung, heart, liver, kidney and spleen tissues

As shown in Figure 3, the expression levels of p16(INK4a) protein in lung, heart, liver, kidney and spleen tissues were significantly higher in the CSE+Dec group and CSE group than that in the control group (p<0.01). Furthermore, the expression levels of p16(INK4a) protein in lung and heart tissues were significantly higher in the CSE group than in the CSE+Dec group (p<0.01 or p<0.05). There was no difference in p16(INK4a) protein in liver, kidney and spleen tissues between the CSE+Dec group and CSE group (p>0.05). There was no difference in the expression levels of p16(INK4a) protein in heart, liver, kidney and spleen tissues between the control group and Dec group (p>0.05), but in lung tissue (p<0.01). As shown in Figure 3, the expression levels of p16(INK4a) mRNA in lung, heart, liver, kidney and spleen tissues were significantly higher in CSE group than those in the control group (p<0.01). The expression levels of p16(INK4a) mRNA in lung, liver, kidney and spleen tissues were significantly higher in the CSE group than in the CSE+Dec group (p<0.05 or p<0.01). The expression levels of p16(INK4a) mRNA in lung, heart, kidney and spleen tissues were significantly higher in the CSE+Dec group than those in control group and Dec group (p<0.05 or p<0.01). The expression levels of p16(INK4a) mRNA in lung, heart, kidney and spleen tissues were significantly higher in the Dec group than in the control group (p<0.05 or p<0.01).

### Expression levels of Sca-1 protein and mRNA in lung, heart, liver, kidney and spleen tissues

**Figure 4:**
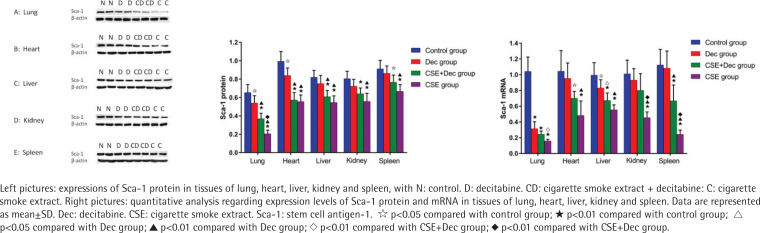
Expression levels of Sca-1 protein and mRNA in lung, heart, liver, kidney and spleen tissues

As shown in Figure 4, the expression levels of Sca-1 protein in lung, heart, liver, kidney and spleen tissues were significantly lower in the CSE+Dec group and CSE group than in the control group (p<0.01 or p<0.05). Furthermore, the expression levels of Sca-1 protein in lung tissue were significantly lower in the CSE group than in the CSE+Dec group (p<0.01). There was no difference in the expression levels of Sca-1 protein in heart, liver, kidney and spleen tissues between the CSE+Dec group and CSE group (p>0.05). The expression levels of Sca-1 protein in lung and heart tissues were significantly lower in the Dec group than in the control group (p<0.05). There was no difference in the expression levels of Sca-1 protein in liver, kidney and spleen tissues between the control group and Dec group (p>0.05). As shown in Figure 4, the expression levels of the Sca-1 mRNA in lung, heart, liver, kidney and spleen tissues were significantly lower in CSE group than in the control group (p<0.01). The expression levels of Sca-1 mRNA in lung, kidney and spleen tissues were significantly lower in the CSE group than in the CSE+Dec group (p<0.05 or p<0.01). The expression levels of Sca-1 mRNA in lung, heart, liver and spleen tissues were significantly lower in the CSE+Dec group than in the control group (p<0.05 or p<0.01). The expression levels of Sca-1 mRNA in heart, liver, kidney and spleen tissues were significantly lower in CSE group than in the Dec group (p<0.01). There was no difference in the expression levels of Sca-1 mRNA in heart, kidney and spleen tissues between the control group and Dec group (p>0.05).

### Expression levels of eNOS protein and mRNA in lung, heart, liver, kidney and spleen tissues

**Figure 5:**
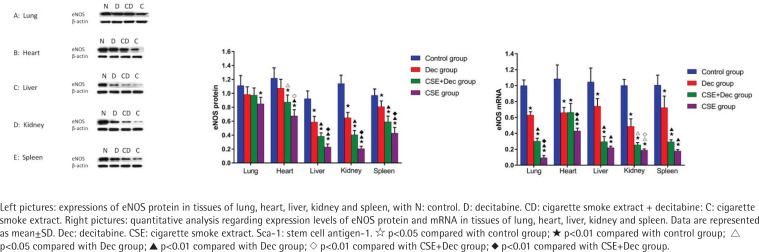
Expression levels of eNOS protein and mRNA in lung, heart, liver, kidney and spleen tissues

As shown in Figure 5, the expression levels of eNOS protein in lung, heart, liver, kidney and spleen tissues were significantly lower in CSE group than in the control group (p<0.01). The expression levels of eNOS protein in heart, liver, kidney and spleen tissues were significantly lower in CSE group than in CSE+Dec group (p<0.05 or p<0.01). The expression levels of eNOS protein in heart, liver, kidney and spleen tissues were significantly lower in CSE+Dec group than those in the control group and Dec group (p<0.05 or p<0.01). The expression levels of eNOS protein in liver, kidney and spleen tissues were significantly lower in Dec group than in the control group (p<0.01). There was no difference in the expression level of eNOS protein in heart tissue between the Dec group and control group (p>0.05). There was no difference in the expression level of eNOS protein in lung tissue among the control group, Dec group and CSE+Dec group (p>0.05). As shown in Figure 5, the expression levels of eNOS mRNA in lung, heart, liver, kidney and spleen tissues were significantly lower in Dec group, CSE+Dec group and CSE group than in control group (p<0.01). The expression levels of eNOS mRNA in lung, heart and kidney tissues were significantly lower in CSE group than those in CSE+Dec group and Dec group (p<0.05 or p<0.01). The expression levels of eNOS mRNA in lung, liver, kidney and spleen tissues were significantly lower in the CSE+Dec group than in the Dec group (p<0.01 or p<0.05).

## DISCUSSION

The present study demonstrated that in the emphysema animal model induced by CSE, the histomorphology of lung tissue was significantly changed, while other organs including heart, liver, kidney and spleen exhibited normal structures and histomorphology. The concentrations of SCF, CyclinD1 and SOD in serum were decreased in the animal model of emphysema induced by CSE. Dec could alleviate the CSE-induced emphysema in histomorphology and alleviate the decrease of Cyclin D1 and SOD caused by CSE; the results of the present study are consistent with our previous study^3^. More importantly, not only in lung tissue, but also in heart, liver, kidney and spleen tissues, the expression levels of p16 (INK4a) protein and mRNA were significantly increased, and the expression levels of Sca-1, eNOS protein and mRNA were significantly decreased in the CSE-induced emphysema animal model. Furthermore, Dec could partly alleviate the changes caused by CSE and the degree of alleviation varied from organ to organ. The results firstly confirmed that there was multiple organ aging in the animal model of emphysema induced by CSE, in another words, CSE could induce multiple organ aging; a methylation mechanism may be involved in the progress of CSE-induced multiple organ senescence and the degree of involvement of methylation mechanism may vary from organ to organ.

The aging of the tissue could be reflected in the tissue histomorphology. In the present study, the histomorphology of lung tissue was significantly changed in CSE-induced emphysema animal model, while the heart, liver, kidney and spleen tissues exhibited normal structure and histomorphology in CSE-induced emphysema animal model. Clinically, many patients with COPD are not complicated with dysfunction of heart, liver and kidney, suggesting that there is a possibility that the aging of the lung precedes other organs, the sensibility and affinity of the lung to CSE are higher than in other organs.

Aging can be determined not only by structure and histomorphology, but also by specific indicators. Usually, the abnormal baseline of specific indicator occurs prior to the abnormal in structure and histomorphology, and also prior to the dysfunction of an organ. SCF is an important growth factor in the hematopoietic system, it acts on the earliest hematopoietic stem cells and hematopoietic progenitor cells, promotes the proliferation and differentiation of hematopoietic cells and regulates the growth and development of hematopoietic cells. Cyclin D1 acts on G1 phase and interacts with many proteins to promote cells to enter S phase. SOD could eliminate excess oxygen free radicals and their derivatives so as to protect cells from damage and maintain normal metabolism. Appropriate concentration of SOD in the body is essential for keeping healthy, and slowing down the process of aging. The antibody p16(INK4a) is one of the best aging biomarkers; suppressed in early embryogenesis and progressively induced during aging. Sca-1 is a glycosylated phosphatidylinositol anchored protein and a cell surface marker found on hematopoietic stem cells (HSCs), is usually associated with stem cells or progenitor cells proliferation and self-renewal, and is a marker of youth. eNOS is the key enzyme that catalyzes the production of NO, an endogenous signal molecule which maintains vascular homeostasis including blood pressure homeostasis, vascular permeability, tension regulation and the hypoxic compensatory mechanism.

Lung aging occurs in lung diseases such as COPD, idiopathic pulmonary fibrosis (IPF) and acute lung injury. Mesenchymal stem cells derived from induced pluripotent stem cell may own anti-apoptotic/pro-proliferative capacity *in vivo* and in CS-induced airway cell partly through paracrine secretion of SCF^7^. Hyperoxia decreases the expression of cyclinD1, which induces proliferation restriction and apoptosis of primary type II alveolar epithelial cells in the lung^8^. Improved SOD expression in mice with COPD protects lung tissues from oxidative stress and inflammation, and inhibits apoptosis of pulmonary endothelial cells^9^. The results of the expression levels of p16(INK4a), Sca-1 and eNOS in the present study are consistent with our previous studies^3,10^ and indicate that the body has damage repair ability related to senescence. Cardiovascular aging and heart failure may be due to the limited cardiac regeneration capacity which is mainly caused by excessive oxidative stress and chronic low-grade inflammation^11^. SCF reduces cardiac myocyte apoptosis in an old heart as well as in a young heart^12^. Increased expression of Cyclin D1 activates multiple cardiac proliferative pathways, promotes adult cardiomyocytes proliferation and preserves cardiac performance after myocardial infarction. Genetic deletion of Sca-1 could result in early-onset of cardiac contractile deficiency as well as age-associated hypertrophy^13^. In a rat liver injury model induced by thioacetamide, SCF promotes liver tissue repair^14^. After liver irradiation, the level of CyclinD1 in the liver increased in mice, which promotes the regeneration of hepatocyte^15^. The increased SOD level could protect mice from acute alcoholic liver injury^16^. Sca-1+ endothelial cells grow aggressively and play an important role in the recovery of severely damaged liver^17^. SCF protects cells from apoptosis in acute kidney injury^18^. Decreased cyclin D1 causes apoptotic characteristics in the ultrastructure of kidney^19^. Sca-1 plays an important role in renal epithelial cell homeostasis and in the recovery of renal function damaged by ischemic acute kidney injury^20^. Upregulating expression level of eNOS ameliorates kidney fibrosis^21^. Endothelial and mesenchymal-like cells secrete SCF to enhance erythropoiesis in the spleen of murine embryos^22^. Reduced SOD level in spleen was associated with the increased risk for cancer in rats^23^. When 10% of total blood of body is lost, the number of Sca-1(+) cells increased in the spleen of mice, which improved the hematopoietic function of the spleen^24^. Upregulating level of eNOS/NO attenuates the inflammatory response in spleen^25^. Cigarette smoke reduces the activity of immunoproteasome and histocompatibility complex class I-mediated antigen in spleen, which damages the immune response in COPD patients and results in cigarette smoke-induced emphysema in mice^26^. Patients with COPD have increased risk for hepatobiliary diseases, asymptomatic elevations of hepatic transaminases and renal complications^27^. Emphysema, an independent common risk factor for kidney disease, is associated with kidney dysfunction in smokers. Intraperitoneal injection of CSE not only causes emphysema, pulmonary parenchymal apoptosis, but also results in injury of cardiac and skeletal muscles in mice^28^. In the present study, although the histomorphology of heart, liver, kidney and spleen tissue was unchanged in CSE-induced emphysema animal model when compared to the control, the concentrations of SCF, CyclinD1, SOD in serum were decreased, and the change trend of the expression levels of p16 (INK4a), Sca-1 and eNOS in heart, liver, kidney and spleen tissues caused by CSE were consistent with what were seen in lung tissue, suggesting that the capacities of regeneration and repairment of the five organs described above were decreased, and not only lung tissue but also heart, liver, kidney and spleen tissues were in the progress of aging in CSE-induced emphysema animal model.

Epigenetic studies are helpful for illuminating some pathophysiological mechanisms of some diseases. Dec is a deoxynucleoside analogue of cytidine, in which the carbon 5 position of the pyrimidine ring is replaced by nitrogen. It is an inhibitor of DNA methyltransferase and could trigger demethylation, leading to gene reactivation. It was verified that Dec could protect against CSE-induced emphysema in animal models^3^. In the present study, the decreased concentration of CyclinD1 and SOD in serum, the increased expression of p16(INK4a), the decreased expression of Sca-1 and eNOS caused by CSE in multiple organs could be partially alleviated by Dec, which suggests that the mechanism of DNA methylation might be involved in the progress of CSE-induced multiple organ senescence. This conclusion is consistent with a previous study^29^. As the degree of alleviation resulting by Dec described above varies from organ to organ, the degree of involvement of methylation mechanism may vary from organ to organ. The differences between the Dec group and control group may be due to the side effect of Dec on the organs in normal individuals.

## CONCLUSIONS

The present study demonstrated that additionally to the aging of the lung tissue in the emphysema animal model induced by CSE, the tissues of the heart, liver, kidney and spleen tissues were also in the progress of aging, although these organs exhibited normal structure and histomorphology except lung. Dec could partly alleviate the changes caused by CSE and the degree of alleviation varies from organ to organ. The results of the present study provide novel understanding and perspective in the pathogenesis of emphysema or COPD and in the systematic impact of cigarette smoking on the human body.

## Data Availability

The data supporting this research are available from the authors on reasonable request.
